# Considerations for emergency department virtual triage

**DOI:** 10.1177/08404704241298643

**Published:** 2024-11-08

**Authors:** Laila Nasser, Emily Morris, Irene Mathias, Justin N. Hall

**Affiliations:** 1 71545 Sunnybrook Health Sciences Centre, Toronto, Ontario, Canada.; 2 6396 Oxford University, Oxford, England, United Kingdom.; 3 University of Toronto, Toronto, Ontario, Canada.

## Abstract

Health leaders are increasingly interested in harnessing Artificial Intelligence (AI) to remotely conduct virtual triage for Emergency Department (ED) patients. This study explores equity considerations and patient attitudes to virtual triage in a Canadian ED. A cross-sectional study surveyed 150 ED patients, with 32 additional patients interviewed in-depth. Descriptive statistics and qualitative descriptive methodology were employed: 84.7% of patients would consider virtual triage, 71.3% were comfortable following advice to seek alternate care, including their General Practitioner or virtual ED. Approximately 38.2% of patients >60 years would require assistance using virtual triage, with confidence in using technology to direct care decreasing with age. Thematic analysis revealed five key themes: value of decision support; care access expectations; technological literacy demographics; trust in AI; and confidentiality. In conclusion, virtual triage is a viable and promising tool if barriers to technological literacy are addressed, and tools are endorsed by health providers and patients.

## Introduction

Health leaders are increasingly interested in harnessing Artificial Intelligence (AI) to remotely conduct virtual triage for Emergency Department (ED) patients. A large academic health sciences centre in Toronto is considering if virtual triage would be a safe option for patients to remotely self-triage and have a machine learning algorithm direct them to a safe avenue of care depending on their level of acuity (i.e., to their family doctor, walk-in clinic, virtual ED, or the in-person ED). Currently, patients who attend an ED are triaged by a nurse using a standardized tool such as the Canadian Triage and Acuity Scale (CTAS), and then seen by a licensed independent practitioner.^1^ Virtual triage entails patients remotely inputting their symptoms into an electronic tool, which then uses an AI-based algorithm to predict their level of acuity, and provide direction on the level of care required. AI-supported virtual triage may increase efficiency in congested emergency departments, more accurately predict patient outcomes including the need for admission, and reduce subjectivity and personal biases in assigning acuity scores.^2-4^ While virtual triage is an emerging field, some proprietary algorithms have shown much promise to safely identify patient acuity levels with decreased subjectivity than nurse-led triage.^2,5,6^ However, among these new tools, triage accuracy is widely variable ranging from 48.8% to 90.1%, with up to 15% under-triage in some studies (comparable to healthcare provider phone triage).

This study aimed to explore patient attitudes toward virtual triage and identify potential equity gaps for hospital leadership to consider. The primary research question was to understand what proportion of patients would consider using a virtual triage tool. The secondary question investigated which demographic sub-groups would face significant barriers to using a virtual triage tool.

## Methods

A multi-method survey was chosen to explore patient attitudes toward virtual triage and obtain quantitative data on demographic groups likely to use it. In May 2023, we selected 150 patients based on our population size of 65,000 annual ED visits to achieve a 95% confidence interval with a 10% margin of error.^7^ Subsequently, 32 additional one-on-one in-depth interviews were conducted with the same questions to explore the reasoning behind participant responses. A multi-method survey was chosen as it is recommended to perform surveys with both quantitative and in-depth qualitative components for reliable outcomes.^8^ The population was selected by convenience sampling in the ED waiting room until saturation of themes was achieved. Interviewers carried iPads to assist patients inputting their answers on Google Forms. Qualitative descriptive methodology was chosen for the second study phase to understand the participants’ perspectives and unique truths.^9^

### Study design

The Unified Theory of Acceptance and Use of Technology 2 (UTAUT2) is a model used in technology acceptance research focusing on six elements (performance expectancy, effort expectancy, social influence, facilitating conditions, hedonic motivation, price value, and habit) that influence a person’s intention to use technology and the actual use behaviour.^10^ This model served as a framework to create our 19-question survey. Six questions address patient demographics. Thirteen questions address patient attitudes toward virtual triage, technology use in healthcare, and barriers to technology use.

### Inclusion criteria

To participate, patients had to be adults (18 years or older) and speak English or have a translator present.

### Data collection and analysis

We surveyed 150 patients, with a further 32 interviewed in-depth. Interviewers had no prior relationship with the patients, nor were they involved in their care. Quantitative analysis was used for multiple-choice questions with results displayed as descriptive statistics. Thematic analysis was completed for open-ended questions using qualitative descriptive methodology as an iterative process whilst interviews were ongoing to observe for saturation of themes; two sets of coding were completed, the first to identify emerging concepts and the second to focus on salient themes that were then identified by grouping relevant categories of codes. All interviews were completed with no questions left blank once initiated.

## Results

### Demographics

The demographic breakdown of participants is displayed in Table 1. Participants tended to be older (51.3% over age 60), female (56.7%), and have higher incomes (28% over $100,000 per year household income). English was the preferred language of most (94%) and 57.3% were born outside Canada.

**Table 1.** Survey participants’ demographics.

	Number of patients (%)	
	Initial survey n = 150	In-depth interviews n = 32
Age		
<20	3 (2%)	2 (6.3%)
20-29	13 (8.7%)	5 (15.6%)
30-39	19 (12.7%)	0 (0%)
40-49	22 (14.7%)	9 (28.1%)
50-59	16 (10.7%)	5 (15.6%)
60-69	24 (16%)	2 (6.3%)
70-79	35 (23.3%)	6 (18.8%)
≥80	18 (12%)	3 (9.4%)
Gender		
Female	85 (56.7%)	18 (56.3%)
Male	54 (43.4%)	14 (43.8%)
Non-binary, transgender, and prefer not to say	0 (0%)	0 (0%)
Preferred language		
English	141 (94%)	32 (100%)
Other (Mandarin, Russian, Cantonese, French, Ukrainian, Arabic, and Tagalog)	9 (6%)	0 (%)
Ethnic/Racial group		
White - North American or European	77 (51.3%)	21 (65.8%)
East Asian	23 (15.4%)	5 (15.6%)
South Asian	20 (13.3%)	5 (15.6%)
Black	14 (9.3%)	1 (3.1%)
Middle Eastern	7 (4.7%)	0 (0%)
Other	5 (3.3%)	0 (0%)
Prefer not to say	4 (2.7)	0 (0%)
Canadian birthplace		
Yes	64 (42.7%)	17 (53.1%)
No	86 (57.3%)	15 (46.9%)
Household income		
$0-$14,999	4 (2.7%)	0 (0%)
$15,000-$29,999	7 (4.7%)	1 (3.1%)
$30,000-$59,999	22 (14.7%)	4 (12.5%)
$60,000-$99,999	37 (24.7%)	6 (18.8%)
≥$100,000	42 (28%)	14 (43.8%)
Prefer not to say	38 (25.4%)	7 (21.9%)

### Use of virtual triage

When asked, “If offered the option to conduct an electronic triage process from home that would help direct your care (i.e., direct you to virtual care, a walk-in clinic, the emergency department, or 9-1-1 based on your complaint), would you consider using this electronic tool?”, 84.7% (n = 127) of participants said yes, 12.7% (n = 19) said no, and 2.6% (n = 4) had caveats to using the tool. The remaining feasibility study results are framed under the UTAUT2 domains on which the survey was built. Descriptive statistics are displayed; further statistical analysis was not completed as the study was not powered to do so.

### Performance expectancy

**Figure 1.**
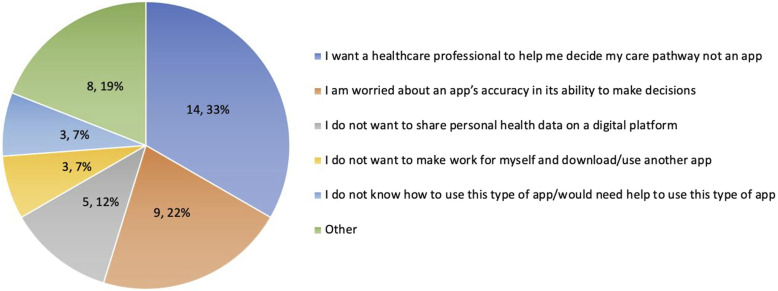
Patient reasons for not wanting to use virtual triage (n = 42).

To ascertain patient expectations from their triage experience, we asked what their reasoning would be to not use a virtual triage tool. Of patients surveyed, 81 (54%) said they would use the tool, 24 (16%) had no comment, and the remaining 42 (28%) provided the responses in Figure 1.

### Effort expectancy

When asked about limitations that would prevent accessing a virtual triage platform, 83% (125 patients) reported no barriers. Of those reporting barriers, 2% (3 patients) had no access to electronic devices, 1.3% (2 patients) had no or unreliable internet access, and 2.7% (4 patients) had both issues.

To gauge the effort patients would expend on using a triage app, we explored actions patients would be willing to take and potential barriers (i.e., downloading an app or logging onto a web site). Overall, 84% (126 patients) were willing to log onto a web site; 62.7% (94 patients) were willing to download an app. Four patients indicated they would prefer to use an existing app such as “MyChart,” the app currently used by patients to access their electronic health record and view their laboratory and imaging results.

### Social influence

We assessed how social context affects virtual triage use, particularly whether patients would need assistance from family, friends, or caregivers. We stratified the data by age, dichotomizing seniors (>60 years) vs. the rest of the adult population, given the higher proportion of seniors in our ED compared to the Canadian population.^11^ Under age 60 (73 patients), only 6.9% said they would require assistance to use virtual triage. However, of patients aged 60 and older (77 patients), 38.2% said they would need assistance.

### Facilitating conditions

**Figure 2.**
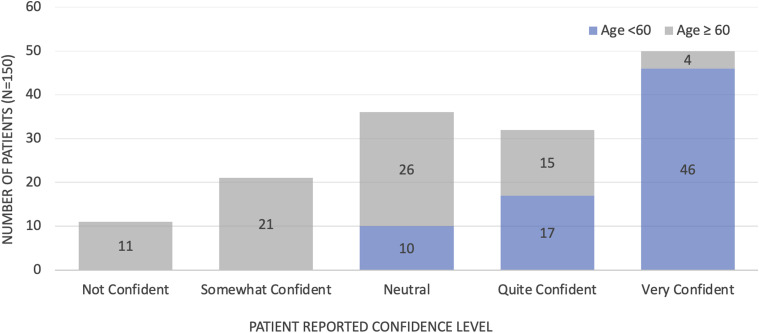
Patient reported confidence in their ability to use technology to direct care.

Trust in technology, belief in its potential benefit, and confidence in its use are key to the success of a virtual triage tool. Patients were asked if they would be comfortable following a virtual triage tool’s advice to seek care in the Virtual ED or with their family doctor instead of the ED; 71.3% (107 patients) said yes, 14.0% (21 patients) said no, and 14.7% (22 patients) said other, indicating their trust would depend on the severity of their symptoms and whether they agreed with the recommendation. We also asked whether patients believed technology could improve ED experience, with 79.3% (119 patients) responding positively. Participants were then asked on a Likert scale their confidence level in their ability to use technology to direct their care, the results of which are displayed in Figure 2.

### Hedonic motivation

To assess hedonic motivation, patients were asked whether they feel satisfaction from replacing in-person tasks with technology-based alternatives. In response, 30.7% (40 patients) answered positively; 58% (88 participants) answered negatively, with the remainder stating it would depend on the circumstances.

### Price value

As a government-funded health system, ED care in Canada is generally free for all persons with a valid health card.^12^ Certain aspects, however, require patients to pay or have separate insurance. To assess the desire for such a tool, we asked how much patients would be willing to pay for an app to facilitate triage from their homes and stratified by household income. A majority (78%, n = 104) only wanted a free app regardless of income. Of the 20% (n = 28) willing to pay up to $10, almost half had a household income over $100,000.

### Habit

Habit has a large influence on whether people will access a specific type of technology.^12^ When asked whether patients have ever used a symptom checker app to assist in deciding whether or where to seek healthcare, 70.7% (106 participants) said they had never used such an app, and 26% (39 participants) indicated that they had, with the remaining 4 participants (3.3%) stating they had tried to use Google or telephone triage available in other countries for this purpose.

## Thematic analysis

Using qualitative descriptive methodology, results are displayed based on how they were extracted from the data and not based on a particular framework. Participant quotations were reviewed for potential patterns that represented themes.

### Theme 1: Value of decision support

Patients feel that virtual triage can be used to validate uncertainty when urgency is unknown, but not in high acuity conditions. Several patients had similar opinions to Participant 23B who stated: *“…if they were new symptoms and I was unsure of their severity, then I would use the tool. It would be most useful for the grey area symptoms where I wouldn’t know… how urgently I need care.”*

### Theme 2: Expectation of efficient access to care if virtual triage is used

Given the long wait times throughout Canadian EDs currently, participants were eager to find solutions that may make the process more efficient.^13^ Several patients cited they would use it if it helped them get “*faster”* care. Virtual triage aims to help with wait time levelling across the system as demonstrated by 28B: *“if it could tell me… which ED has the shortest wait, I would probably use it more*.*”*

### Theme 3: Demographic influence on technological literacy

The theme of effort expectancy was prominent in participants over the age of 60, many of whom discussed physical and social barriers to being able to use a virtual triage tool. Some cited their *“loss of sight”* (27B), having *“no one around [to help]”* (30B) and existing difficulty using technology in the ED such as *“struggling with the registration kiosk to check in”* (30B). Not all elderly patients reported issues with technological literacy, and participant 25B stated “*technology allows us to achieve the same things, faster and with fewer resources…you just have to ensure [it] can meet every patient’s needs*”. Many patients over age 60 however felt like participant 30B, who said “*Technology seems to complicate things unnecessarily and is making it harder for me to access my care. It seems to be leaving my generation behind*.”

### Theme 4: Trust in virtual triage tools

Social influence is a major driver in whether patients would consider using a virtual triage tool. Specifically, patients want to know the tool is trusted by their physicians and families. Participant 6B stated *“if it were hospital recommended and if lots of people I knew had used it… I would be more likely to [use it]”*. Similarly, Participant 23B stated it would depend on *“how good the evidence base was… if it was clearly shown to be safe and recommended by doctors then I would follow the advice.”*

Some patients describe lacking trust in the accuracy of virtual triage without an in-person assessment or healthcare assistance using the tool. Many patients documented unique circumstances and comorbidities as reasons for wanting in-person triage. For example, Participant 24B worried their stroke would have been missed by virtual triage due to vague symptoms, and participant 25B worried their terminal cancer warranted in-person targeted advice.

Trust also depended on how different the tool’s advice would be from what patients expected. There were also patients who were ready to *“trust the judgement of a tool developed by my doctors much more than my own”* (22B) and understood that *“[technology] can malfunction, but I don’t think human error is too different*” (23B).

### Theme 5: Maintaining confidentiality

Several patients described data privacy as a potential concern with using a virtual triage tool, but the majority said it would not hinder use if privacy could be ensured.

## Discussion

There was strong support for the use of a virtual triage tool to guide potential ED patients to alternative care locations. Exploring performance expectancy highlighted that virtual triage could validate uncertainty when urgency is unknown, but not in high acuity conditions. Patients hesitant to use a virtual tool wanted a healthcare practitioner to help them decide their care pathway; a “help” function to connect users with a real-time provider may be prudent to alleviate some patients’ lack of trust in the accuracy of a virtual tool.

UTAUT2 is the most widely cited framework to evaluate a technology’s acceptability; thus, our results suggest that if a virtual triage tool was available, it would be accepted.^14^ Results around performance expectancy were mostly positive and this element is a direct determinant of behavioural intention and has shown the largest effect size of all six elements.^15^ However, we learned that patients expect improved efficiency in accessing care with the use of a triage tool, which is possible if used to expedite registration or direct patients to a faster source of care.

The findings related to the senior demographic are noteworthy, with 38.2% of seniors saying they would require assistance using virtual triage compared to 6.9% of other adults. The potential challenges related to technological literacy in the elderly population is again demonstrated by their confidence in ability to use technology to direct care clearly decreasing with age. For optimal uptake, a triage tool must be piloted with the elderly demographic during development to ensure equitable use. Given that patients were more willing to use a web site (84%) than an app (62.7%), simplifying access to virtual triage is equally important.

Addressing, socio-economic barriers to triage tool access, 7.4% of participants were considered low income (household income less than $30,000) per Canadian standards.^16^ Patients of lower socio-economic status have different healthcare needs and decreased ability to access care, so suggesting alternate care pathways may not prove useful when the ED is often the easiest access point to the healthcare system.^17^ Access to technology itself does not appear to be a limiting factor, as most of this demographic have cellular phone access with 94% owning a device and 85% using a cellular phone daily.^18^

## Limitations

There are multiple limitations to this study. Self-reported data may be influenced by participant interpretation, central tendency bias and motivation to participate.^19^ Although targeting our audience of interest, only those well enough to wait in the ED waiting room were interviewed. Furthermore, the Hawthorne effect may have influenced favourable responses as patients were interviewed in the area they were waiting to be treated in; there was an attempt to minimize this by having non-Sunnybrook staff act as interviewers. While the intention was to understand feasibility at this site, single-centre results may not be generalizable to other EDs.

Another limitation involves language proficiency. Only 6% of the initial survey participants had a preferred language other than English and none in the in-depth surveys. Patients with limited command of the English language may face difficulty communicating their symptoms, affecting the accuracy of a virtual triage tool, which would not necessarily be reflected in our results.^17^ However, a multi-lingual tool could improve access for patients in the largely anglophone system.

## Conclusion

A virtual triage tool is a viable option for the current study population. However, careful consideration must be made by health leaders with respect to tool platform and design, to help reduce barriers in accessing technology to direct care. The tool must also be endorsed by healthcare providers and patient families for optimal uptake.

## Data Availability

The datasets generated and/or analyzed during the current study are available from the corresponding author on reasonable request.
